# Evaluation of a Digital Intervention for Monitoring and Improving Medication Adherence Among Real-World e-Consumers of HIV Preexposure Prophylaxis in China: Protocol for a Randomized Controlled Trial

**DOI:** 10.2196/92750

**Published:** 2026-06-29

**Authors:** Jingtao Zhou, Qingyu Li, Jiayin Zheng, Huang Xiao, Yuhang Zhang, Siwen Huang, Chi Ruan, Zhiyi Zhao, Chunqing Lin, Huachun Zou, Xianlong Ren, Houlin Tang, Hui Xue, Sitong Luo

**Affiliations:** 1 Vanke School of Public Health Tsinghua University Beijing, Beijing China; 2 National Institutes for Food and Drug Control Beijing, Beijing China; 3 Department of Psychiatry and Biobehavioral Sciences David Geffen School of Medicine University of California Los Angeles Los Angeles, CA United States; 4 School of Public Health Fudan University Shanghai, Shanghai China; 5 Beijing Center for Disease Prevention and Control Beijing, Beijing China; 6 National Center for AIDS/STD Control and Prevention Chinese Center for Disease Control and Prevention Beijing, Beijing China; 7 HeHealth Beijing, Beijing China; 8 Institute for Healthy China Tsinghua University Beijing, Beijing China

**Keywords:** HIV/AIDS, preexposure prophylaxis, mobile health, mHealth, intervention, preexposure prophylaxis adherence, PrEP adherence

## Abstract

**Background:**

Preexposure prophylaxis (PrEP) is a key biomedical HIV prevention strategy that relies heavily on adherence for optimal effectiveness. In China, most PrEP users purchase their medication online, making it challenging to monitor and support adherence effectively.

**Objective:**

This protocol aims to describe a digital intervention developed to monitor and improve the medication adherence of real-world e-consumers of PrEP and an evaluation plan assessing its acceptability, feasibility, and effectiveness.

**Methods:**

The Real-Time Monitoring and Precision Intervention for HIV PrEP Adherence (REMOTE) trial is a parallel-group, open-label, online-delivered, active-controlled, and stratified randomized controlled trial conducted among 430 e-consumers of PrEP (320 event-driven regimen users and 110 daily regimen users) in China. People who have purchased PrEP online, have taken PrEP in the previous 3 months, and plan to continue for the next 6 months will be stratified by regimen type and randomized into control and intervention groups at a 1:1 ratio. A WeChat-based digital platform will deliver the REMOTE intervention. The monitoring module follows ecological momentary assessment principles. The intervention design is guided by the stages of change theory, tailoring strategies to different nonadherence risks based on monitoring results. Intervention components include low-risk (health education and an artificial intelligence chatbot), medium-risk (peer forum and admonitory education), and high-risk (customized reminders and physician counseling) strategies. The control group will use a simplified platform with only real-time monitoring. The primary outcome is the proportion of participants achieving optimal adherence for 6 months, assessed via real-time monitoring and validated via surveys at baseline and the 1-, 3-, and 6-month follow-ups. Secondary outcomes include PrEP adherence knowledge, self-efficacy, risk perception, adherence barriers, stigma, and social support, measured via surveys. Intention-to-treat analysis will be conducted.

**Results:**

Funding for the study was approved in March 2024. Ethics approval for the study was granted in July 2024. The pilot trial was completed in November 2025. Baseline data collection commenced in January 2026. By February 5, 2026, recruitment and baseline data collection were completed, with 448 participants enrolled. The data have not been viewed by the research team. The intervention is currently ongoing, and the study is expected to conclude in August 2026. Results are anticipated to be published in early 2027.

**Conclusions:**

The REMOTE trial pioneers a real-time monitoring and precision intervention for e-consumers of PrEP. Leveraging technology and ecological momentary assessment, it delivers a personalized, real-time intervention that is crucial for adherence. The findings could significantly impact future HIV prevention strategies.

**Trial Registration:**

Chinese Clinical Trial Registry ChiCTR2400088278; https://www.chictr.org.cn/showproj.html?proj=236414

**International Registered Report Identifier (IRRID):**

DERR1-10.2196/92750

## Introduction

HIV and AIDS continue to be a severe public health threat worldwide [1]. Preexposure prophylaxis (PrEP) is a highly effective biomedical strategy for preventing HIV infection, and it is strongly recommended for individuals at higher risk of HIV acquisition by the World Health Organization [2]. PrEP can reduce the risk of HIV transmission significantly across all populations when taken as instructed [3]. However, the effectiveness of PrEP heavily depends on optimal medication adherence. Previous studies have shown that, for every 10% reduction in PrEP adherence, the effectiveness of PrEP is reduced by 13% [4,5]. Oral PrEP offers 2 medication regimens currently: daily PrEP and event-driven PrEP [6]. For daily PrEP, users must take 1 pill every 24 hours, starting at least 7 days before engaging in high-risk behavior [7]. For event-driven PrEP, known as the “2-1-1” protocol, users are required to take 2 pills 2 to 24 hours before anticipated sexual activity, followed by 1 pill 24 hours after the initial dose and another pill 48 hours after [8]. A large body of literature shows that suboptimal PrEP adherence is common worldwide, with the reported prevalence of good adherence ranging from 38% to 97%, where the proportion of good adherence among daily users is significantly higher than that among event-driven users [9-12].

In China, PrEP was officially approved by the government in 2020 [13], and most users are young men who have sex with men of Han ethnicity, with approximately 70% obtaining their medication through online platforms in real-world settings [14-16]. On these platforms, people who are willing to purchase PrEP are first required to complete an online screening questionnaire for PrEP eligibility (eg, recent HIV testing results and contraindications to PrEP). A physician then reviews the screening questions remotely and issues an electronic prescription if the clients meet PrEP eligibility. In China, obtaining PrEP through traditional clinical channels requires visiting designated hospitals, undergoing rapid HIV testing, and receiving a physician’s risk assessment, which can be time-consuming. In contrast, online purchasing platforms allow users to complete the screening questionnaire to obtain PrEP remotely, offering a more convenient and accessible alternative. Moreover, compared to traditional channels, e-commerce platforms such as HeHealth, the biggest and most popular internet hospital for selling PrEP in China, occasionally offer promotional discounts, making online access slightly more affordable. Despite the advantages, there are unique challenges for e-consumers of PrEP to achieve optimal medication adherence compared to traditional clinic clients due to the nature of online sales, such as lack of face-to-face communication with physicians and frequent follow-up and counseling regarding their adherence and HIV risk.

Our recent cohort study using surveys showed that the prevalence of optimal adherence (ie, strictly following the 2-1-1 guidelines for event-driven users and taking 1 pill per day for daily users) among 657 e-consumers of PrEP in China ranged from 80.7% to 87.8% for the daily regimen and 38.6% to 44.1% for the event-driven regimen (the main medication regimen chosen by real-world Chinese users). The main factors associated with nonadherence included sociodemographics (eg, younger age), behavioral factors (eg, chemsex and multiple sex partners), and psychosocial factors (eg, forgetfulness, low self-efficacy, and PrEP stigma) [17]. Previous studies have also identified barriers to PrEP uptake and adherence, such as cost, access, and stigma associated with PrEP [11]. Therefore, there is an urgent need to develop interventions to improve medication adherence for real-world PrEP users. Previous intervention research has used electronic pill boxes, mobile phone apps (visually confirming taking the medication), and ingestible sensors to monitor PrEP adherence in a timely manner and developed digital interventions (eg, message reminders and remote counseling) to improve adherence, but their target groups were all offline clinic PrEP users and were mostly daily regimen users [18-20]. As mentioned above, e-consumers and event-driven regimen users account for most real-world PrEP clients in China, and they face unique challenges with respect to medication adherence that need special attention.

To address these gaps, this study aims to develop a digital platform to achieve real-time monitoring of PrEP adherence based on the principles of ecological momentary assessment (EMA); deliver a just-in-time adaptive intervention guided by the stages of change theory (SCT) and transtheoretical model (TTM); and conduct a randomized controlled trial to evaluate the effectiveness, acceptability, and feasibility of the digital intervention. EMA is a sampling approach that gathers repeated, real-time data on participants’ experiences and behaviors in their natural environments and has become increasingly used in health behavior monitoring with the proliferation of smartphones and internet access [21-24]. The SCT posits that behavior change is a continuous process of 5 distinct stages: precontemplation, contemplation, preparation, action, and maintenance; different intervention strategies should be applied for people at different stages [25]. Moreover, by identifying a participant’s stage, the intervention can deliver tailored, stage-specific strategies that build self-efficacy and support sustained adherence. Different stages receive different interventions, forming a continuous process aligned with the user’s risk level and incorporating the 10 processes of change from the SCT. This framework also acknowledges relapse as part of behavior change, allowing for timely, real-time support to re-engage users. By addressing the specific needs of e-consumers, this study not only fills a critical research gap in the Chinese context but also offers valuable insights and practical implications for promoting medication adherence in similar settings across other low- and middle-income countries.

## Methods

### Study Design

The Real-Time Monitoring and Precision Intervention for HIV PrEP Adherence (REMOTE) trial is a parallel-group, open-label, active-controlled, stratified randomized trial being conducted from November 2025 to August 2026. Eligible e-consumers of PrEP have been enrolled, stratified by regimen, and randomized into the intervention and control group at a 1:1 ratio. The intervention group will be asked to use the full version of a WeChat-based digital platform with real-time monitoring and precision intervention functions developed by the research team for 6 months. The control group will access a simplified version of the platform containing only the real-time monitoring function designed for adherence data collection to minimize recall bias for both groups. As the monitoring system may also improve PrEP adherence, it is considered as an active control strategy. All the participants will be asked to complete the baseline and 1-, 3-, and 6-month surveys. This study protocol was developed in accordance with the SPIRIT (Standard Protocol Items: Recommendations for Interventional Trials) checklist.

### Population

People who (1) are aged 18 years and older, (2) have purchased PrEP through HeHealth (the largest internet hospital selling PrEP in China, formerly known as BluedHealth) and passed the required risk assessment and health examination confirming their eligibility for PrEP use at the time of purchase, (3) have taken PrEP in the previous 3 months and have plans to continue taking the medication in the following 6 months, and (4) provide oral informed consent will be included in the study. People who are (1) incapable of communicating and reading and (2) unable to use mobile phones and the WeChat app will be excluded from the study. This exclusion criterion was established to ensure that all participants can comprehend the study materials and effectively engage with the digital platform throughout the study.

To recruit participants, HeHealth staff will distribute recruitment messages containing a QR code linking to the research team’s WeChat account to potentially eligible individuals via the platform’s internal messaging system. Interested individuals may scan the QR code to contact the research team through WeChat, after which eligibility screening based on the inclusion criteria will be conducted by the research staff. All recruitment materials and procedures used by the research team will ensure neutrality, ethical compliance, and the absence of sales incentives. Information on the purpose of the study, specific study procedures, possible risks and discomforts, potential benefits, contact, and the rights of participating individuals will be clearly stated by the recruiters and presented in the electronic informed consent form. Electronic informed consent will be obtained before any data collection. Once recruited into the study, participants will be assigned a unique ID number for logging into the digital platform and responding to the baseline and follow-up electronic questionnaires.

### Sample Size Planning

The sample size was calculated based on the adherence data we obtained from the previous cohort study and the available financial and human resources [17]. Among event-driven users, a sample size of 320 can detect at least a 17.5% difference in the proportion of optimal adherence between the 2 groups assuming that the proportion of optimal adherence in the control group is 45% (estimated proportion of optimal adherence in the control group, *p*_2_=0.45), the rate of loss to follow-up is 20% (not completing the electronic questionnaire sent during follow-up visits), the level of significance is α=.05 (*Z*_α_=1.64), and the statistical power is β=.8 (*Z*_β_=0.84). Among PrEP daily users, a sample size of 110 could detect at least a 17.5% between-group difference assuming a 60% proportion of optimal adherence in the control group (*p*_2_=0.60), a 10% loss to follow-up rate, a significance level of α=.05 (*Z*_α_=1.64), and a statistical power of β=.8 (*Z*_β_=0.84). Therefore, a minimum of 430 participants will be enrolled in this study.

### Randomization and Blinding

**Figure 1:**
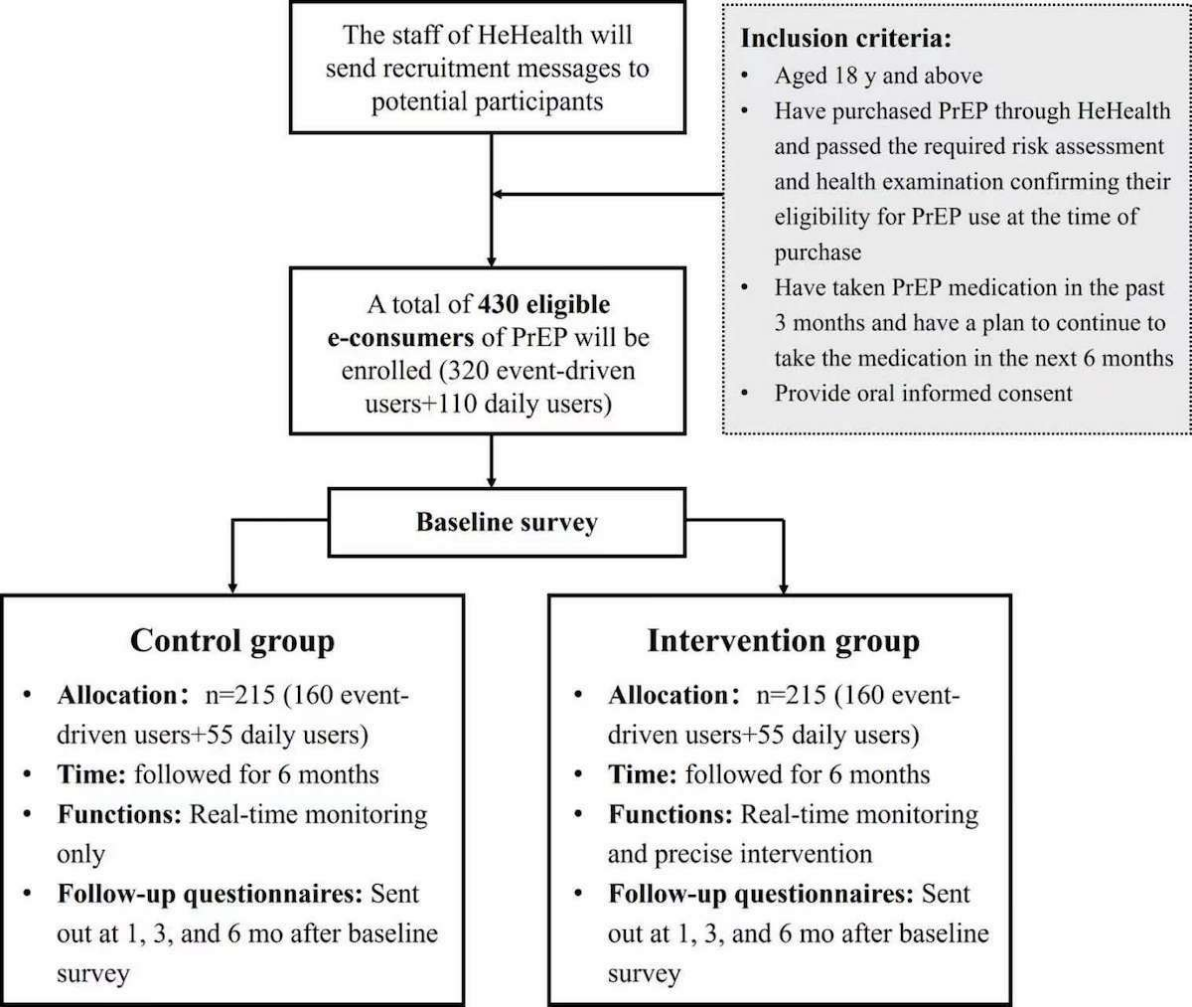
Flowchart of recruitment and follow-up procedures of the study. PrEP: preexposure prophylaxis.

Randomization sequences were generated prior to study initiation by an independent statistician using the R software (R Foundation for Statistical Computing). Participants were stratified by PrEP dosing regimen (daily vs event driven) based on their self-report. Within each stratum, participants were randomized in a 1:1 ratio to the intervention or control group using variable block randomization with randomly permuted block sizes of 4 and 6. As a result, each group will have 215 participants, with 160 event-driven users and 55 daily users (Figure 1). The study adopted an open-label design because the nature of the intervention makes blinding infeasible.

### Control and Intervention Description

#### Real-Time Monitoring

**Figure 2:**
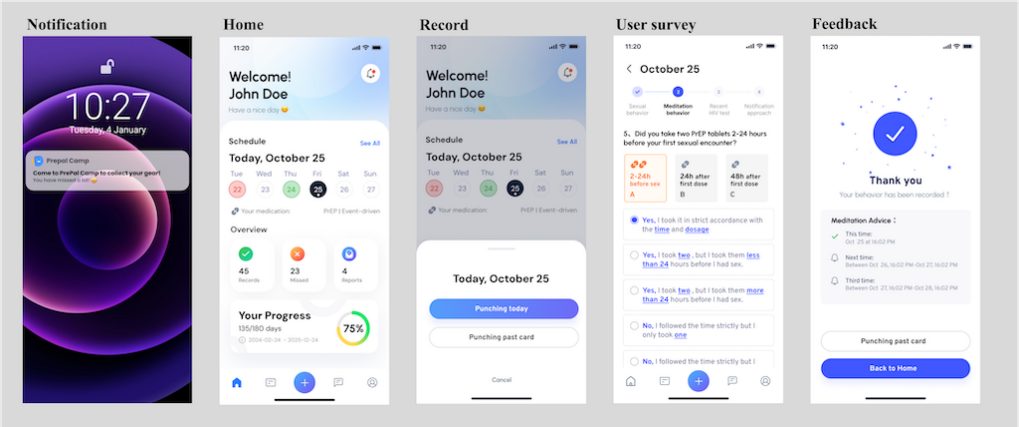
PLP Camp screenshots of the adherence recording journey from the user end.

All the participants in both the control and intervention groups will be invited to use a WeChat-based digital platform named PLP Camp (Figure 2). PLP Camp allows participants to electronically record their PrEP adherence and related sexual behaviors in a timely manner based on the EMA principles of interval- and event-contingent recording. Daily users will receive an SMS text message every 24 hours at a self-predetermined time (interval contingent) to remind them to log on to the platform and record whether they have taken their daily pill in the previous 24 hours. Event-driven users are asked to actively log on to the platform and record their PrEP-taking behavior for each sexual encounter (event contingent). In addition, they will receive an SMS text message every 72 hours at a self-predetermined time (interval contingent) to remind them to log on and record their PrEP-taking behaviors in the previous 72 hours.

According to the records of PrEP-taking and related sexual behaviors, the platform will automatically calculate each participant’s risk level of nonadherence (ie, high, medium, or low risk). The criteria for risk classification are shown in Table 1. The risk level will be assessed every 3 days, with a comprehensive risk assessment report sent to the participants on a weekly basis. Participants who do not report any adherence during each risk assessment period will continue to be assigned their last reported risk level. Moreover, those who are inactive more than 3 times will be kindly reminded to log into the platform via WeChat. The detailed stratification is shown in Table 1. The PLP Camp platform used by the control group (simple version) will only include the function of real-time monitoring with risk assessment.

**Table 1:** Criteria for risk classification of preexposure prophylaxis (PrEP) nonadherence in the Real-Time Monitoring and Precision Intervention for HIV PrEP Adherence trial.

Regimen and risk level			Risk assessment criteria
**Event-driven PrEP**			
	Low risk	Correct medication taken for every sexual encounter	
	Medium risk	Medication taken for every sexual encounter but with partial correct use (ie, incorrect use for at least 1 of the 2-1-1 time points) sometimes or medication not taken for every encounter but with correct use whenever taken	
	High risk	Medication not taken for every sexual encounter and instances of incorrect use	
**Daily PrEP**			
	Low risk	≥80% medication adherence (ie, taking medication on at least 25 out of 30 d)	
	Medium risk	40%-80% medication adherence (ie, taking medication on 12-24 out of 30 d)	
	High risk	<40% medication adherence (ie, taking medication on less than 12 d in a 30-d period)	

#### Precision Intervention Component

**Figure 3:**
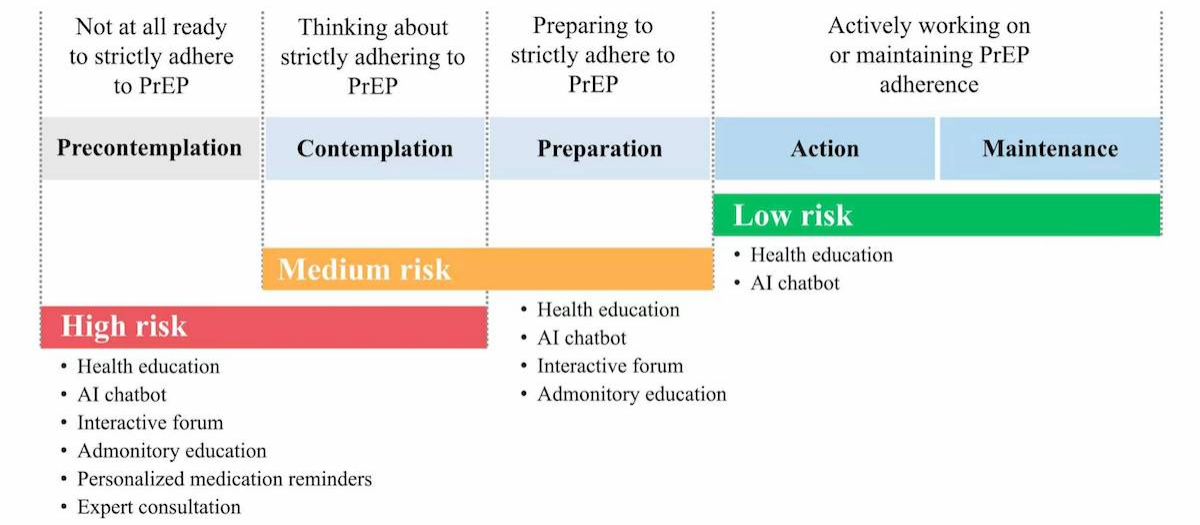
Conceptual framework of the precision intervention. AI: artificial intelligence; PrEP: preexposure prophylaxis.

The full version of the PLP Camp platform used by the intervention group will additionally incorporate precision intervention functions. On the basis of the results of the risk assessment, participants at different risk levels will receive precise and targeted interventions [26]. The intervention is designed based on the assumption that different risk levels of PrEP nonadherence correspond to different stages of change toward optimal adherence, as shown in Figure 3. It is assumed that high risk means that the participants are not ready to adhere to PrEP at all or they are just starting to think about adhering, medium risk means that the participants are starting to think about adhering and preparing to adhere to PrEP, and low risk means that they are currently practicing and maintaining optimal adherence. The intervention was developed through a multistage, theory-informed, and user-centered process. It was grounded in the SCT and TTM, which conceptualize adherence as a gradual behavioral progression. Guided by this framework, we mapped levels of nonadherence risk to corresponding stages of change, thereby enabling delivery of tailored intervention components according to participants’ behavioral readiness [27]. This theoretical alignment ensured that participants with lower readiness received more intensive behavioral support, whereas those with higher readiness received informational and reinforcement support. Different behavior change techniques are used for different risk levels. Each intervention component was aligned with TTM constructs [27].

Low-risk participants accessed weekly health education (eg, HIV and PrEP knowledge, importance and benefits of good adherence, and ways to keep mentally well) and an artificial intelligence (AI) chatbot for counseling (eg, answering questions about correct instructions for PrEP), corresponding to the action and maintenance stages. These modules operationalized counterconditioning by helping participants replace unhelpful behaviors and beliefs with health-promoting alternatives. The AI chatbot provided timely responses to basic questions about PrEP and HIV, reducing anxiety and encouraging proactive problem-solving, whereas the weekly health education delivered evidence-based content and positive narratives that fostered mindfulness, self-efficacy, and constructive attitudes toward adherence.

Medium-risk participants accessed an interactive forum and admonitory education (eg, exemplars and narratives about HIV infection due to PrEP nonadherence), corresponding to the contemplation and preparation stages. The interactive forum mapped to helping relationships, enabling peer exchange and collective problem-solving. The admonitory education module aligned with stimulus control, guiding participants to recognize triggers—such as social situations, travel, or stress—and restructure their environment to support adherence.

High-risk participants received personalized medication reminders (tailored pop-up messages) and expert consultation (web seminars and online individual sessions), corresponding to the precontemplation and contemplation stages. The reminder system reflected consciousness raising by promoting self-observation and awareness of missed doses, whereas the expert consultation module embodied emotional arousal (dramatic relief) by helping participants express emotions related to adherence and receive encouragement and hope through empathetic guidance.

**Figure 4:**
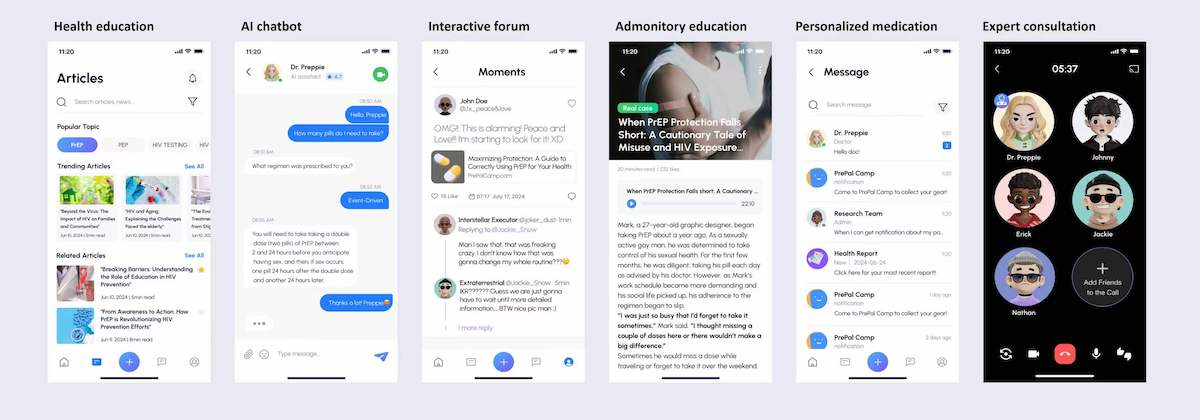
PLP Camp screenshots of each intervention module from the user end. AI: artificial intelligence.

Examples of the platform modules are shown in Figure 4. As the risk level of each participant may change throughout the study period, the intervention modules will be automatically adjusted by the platform based on the participant’s weekly adherence report.

Intervention delivery will be implemented through the PLP Camp platform, with all modules and the check-in function accessible via participants’ mobile devices. Participants will log into PLP Camp through WeChat, ensuring seamless integration with a widely used app. Health education materials are available on the home page and presented in chronological order according to publication time. To promote outreach, the research team will also send health education materials to participants one-to-one via WeChat. The AI chatbot is also accessible from the home page; selecting this feature directs users to an interactive dialogue interface. Admonitory education is provided within the health education section under a dedicated subtab titled “Their Story.” The peer forum is accessible via the “Forum” button on the settings page, where participants can create posts, comment, and engage with others through likes. Personalized medication reminders are automatically triggered when participants are first classified as high risk and can subsequently be accessed through the settings page. Additionally, the “Consult Expert” function, located on the settings page, connects participants to a professional PrEP consultant through a real-time dialogue interface.

### Data Collection

Data for the REMOTE trial will be collected in 2 ways: from the PLP Camp platform and from the baseline and follow-up surveys. The real-time monitoring function of PLP Camp is able to record participants’ PrEP adherence behaviors and related sexual behaviors. The baseline and 1-, 3-, and 6-month follow-up surveys will be self-administered via an electronic questionnaire. Data will be collected by 2 trained research staff members. Sociodemographic information, current PrEP regimen, sexual behaviors, PrEP adherence, PrEP knowledge and perceptions, facilitators of and barriers to PrEP adherence, PrEP stigma and self-efficacy, social support, and other psychosocial variables will be collected (S1, baseline questionnaire; Multimedia Appendix 1). PrEP adherence will be measured by asking participants whether they took PrEP for each sexual encounter and whether they took it strictly as instructed. A PrEP knowledge question will be extracted from the guidelines for taking PrEP [28]. PrEP stigma will be measured using an HIV PrEP stigma scale [29]. PrEP self-efficacy will be measured using an adapted version of the Condom Use Self-Efficacy Scale [30]. Psychosocial variables will be measured using the Patient Health Questionnaire–9, the 2-item Connor-Davidson Resilience Scale, and the 7-item Generalized Anxiety Disorder Scale [31-33]. Follow-up questionnaires will include the same variables, with extra measures of the feasibility and acceptability of the intervention. Feasibility and acceptability will be measured using the Feasibility of Intervention Measure and Acceptability of Intervention Measure scales combined with PLP Camp use and the Intervention Appropriateness Measure scale (S2; Multimedia Appendix 2) [34]. The link to access the questionnaires will be sent to the participants via WeChat, and each survey will take an average of no more than 10 minutes to complete.

### Outcomes

The primary outcome of this study is optimal PrEP adherence over the 6-month study period, defined as a binary outcome (optimal vs suboptimal adherence) and assessed separately for event-driven and daily PrEP users. Adherence will be measured primarily using EMA data collected through the PLP Camp platform version 2.0, whereas survey data collected at baseline and follow-up visits will be used to support interpretation and cross-validation of adherence-related measures.

For daily PrEP users, each PLP Camp check-in will record whether the participant has taken 1 PrEP pill within the preceding 24 hours. Adherence will be calculated as the proportion of days with reported pill intake out of all days under observation during each monitoring period. In constructing this outcome, days without a submitted PLP Camp check-in will be operationally treated as days without reported pill intake. Daily users with adherence of 80% or higher during the relevant monitoring period will be classified as having optimal adherence, whereas those with adherence below 80% will be classified as having suboptimal adherence [4,8,35].

For event-driven PrEP users, each PLP Camp check-in will capture sexual behavior and PrEP use occurring within the reporting interval, defined as the time since the previous check-in up to a maximum of 72 hours. Adherence will be assessed at the level of reported sexual encounters according to the standard 2-1-1 regimen. A reported sexual encounter will be classified as adherent if the participant reports taking 2 PrEP pills 2 to 24 hours before sex, 1 pill 24 hours after the initial dose, and 1 additional pill 48 hours after the initial dose. Event-driven users will be classified as having optimal adherence if all reported sexual encounters during the relevant monitoring period meet these dosing requirements; otherwise, they will be classified as having suboptimal adherence. Intervals without a submitted PLP Camp check-in will be treated as intervals without a reported sexual encounter, and therefore, event-level adherence will be assessed only for reported sexual encounters.

Secondary outcomes will include platform-related implementation outcomes, namely, effectiveness, acceptability, and feasibility of the PLP Camp platform, as well as individual-level determinants related to PrEP adherence, including PrEP-related knowledge, self-efficacy for maintaining adherence, risk perception of HIV infection and PrEP nonadherence, barriers to and facilitators of adherence, PrEP stigma, and social support. These outcomes will be measured using surveys administered at baseline and follow-up visits.

### Statistical Analysis

All analyses will follow the intention-to-treat principle, whereby all randomized participants will be analyzed according to their assigned study group regardless of intervention uptake, regimen changes, or follow-up completeness. Baseline characteristics will be summarized by study group using appropriate descriptive statistics.

The primary analysis will assess between-group differences in optimal PrEP adherence over time. As the primary outcome is binary and repeatedly measured, generalized linear mixed-effects logistic regression models will be used, including fixed effects for study group, time, and the group-by-time interaction and a participant-level random intercept to account for within-participant correlation. Prespecified baseline covariates will be included to improve precision and adjust for residual imbalance. Effect estimates will be presented as odds ratios with 95% CIs.

Adherence outcomes will be constructed according to the prespecified regimen-specific definitions described above. For daily PrEP users, days without a recorded PLP Camp check-in during each monitoring period will be operationally treated as days without reported pill intake based on previous literature [36,37]. For event-driven PrEP users, adherence will be assessed only for reported sexual encounters according to the standard 2-1-1 regimen; intervals without a submitted PLP Camp check-in will be treated as intervals without a reported sexual encounter.

Secondary outcomes will be analyzed using mixed-effects models appropriate to outcome type, with fixed effects for study group, time, and the group-by-time interaction and participant-level random effects. All tests will be 2 sided, with statistical significance defined as *P*<.05. Analyses will be conducted using the R software.

### Missing Data Handling

Missing data are expected to arise primarily from incomplete or absent EMA check-ins and missed follow-up assessments. Prespecified regimen-specific rules used to derive adherence outcomes will be treated as part of the outcome definition rather than as missing data imputation. In the primary analysis, generalized linear mixed-effects models will use all available observed repeated adherence outcomes without requiring complete data at every follow-up time point. This likelihood-based approach allows participants with partially observed follow-up data to contribute to the analysis and is consistent with the intention-to-treat principle under the assumption that missingness depends on observed data.

Sensitivity analyses will be conducted to assess the robustness of the findings to alternative assumptions regarding missing data. First, multiple imputation by chained equations will be applied to missing interval-level adherence outcomes and missing covariate data. The imputation models will include study group, time, prior adherence, baseline characteristics, and other variables associated with adherence or missingness. Estimates from the imputed datasets will be combined using the Rubin rules. Second, complete-case analyses will be performed for comparison. Third, additional sensitivity analyses will examine less favorable assumptions for missing adherence outcomes to assess the extent to which the main conclusions depend on departures from the primary missing data assumption.

### Ethical Considerations

This study was approved by the institutional review board of Tsinghua University (THU01-20240097) on July 4, 2024 and was registered in the Chinese Clinical Trial Registry (ChiCTR2400088278) on August 14, 2024. Electronic consent will be obtained from all participants. Compensation will be provided after each survey completed in the form of electronic vouchers for the HeHealth store. The study findings will be reported in peer-reviewed publications and presented at relevant academic conferences.

## Results

Funding for the study was approved in March 2024. Ethics approval for the study was granted in July 2024. Recruitment and randomization commenced in November 2025. This included identification of eligible participants and randomization of the eligible participants to the control or intervention group until the required sample size was achieved. Baseline data collection commenced in January 2026. By February 5, 2026, recruitment and baseline data collection were completed, with 448 participants enrolled. The data have not been viewed by the research team. The intervention is expected to conclude in August 2026, and results are anticipated to be published in early 2027.

## Discussion

### Expected Findings

The REMOTE trial will evaluate the effectiveness of a digital intervention in improving the PrEP adherence of real-world users who purchase the medication online. The WeChat-based intervention platform incorporates a real-time monitoring function developed based on the EMA approach and precision intervention functions guided by the SCT. The ultimate goal of the REMOTE trial is to provide a feasible and effective tool to help e-consumers of PrEP keep optimal medication adherence to prevent HIV infection. The results of this study will have several important implications for future HIV prevention and broader public health interventions.

First, it addresses a critical gap in current PrEP adherence interventions by specifically focusing on online PrEP consumers, a growing population that faces unique barriers to optimal PrEP use [38,39]. By leveraging AI and EMA, the platform delivers personalized, context-specific interventions in real time, a feature that is crucial for addressing adherence challenges such as forgetting to take the medication or needing timely information and support [40]. Second, this intervention could serve as a model for integrating digital health platforms with real-world adherence monitoring, thereby improving outcomes in other health care areas that require long-term medication adherence (eg, antiretroviral therapy for HIV and chronic disease management) [31-43]. Additionally, the data collected through the intervention platform could offer valuable insights into user behavior and adherence patterns, contributing to future research on HIV prevention and personalized health interventions [44,45]. Policymakers and health care providers may leverage the study’s findings to enhance PrEP programs and expand the use of digital health platforms for preventive interventions, particularly among underserved populations. More importantly, the design and findings of this study may also inform similar efforts in other low- and middle-income countries or regions where online medication procurement is common, but adherence support remains limited. The model could be adapted to fit the sociocultural and infrastructural contexts of other settings, providing a feasible and effective framework for improving adherence to PrEP and other preventive medications delivered through digital channels.

### Strengths and Limitations

While this study offers a promising and innovative approach, several limitations should be acknowledged. First, participants will be conveniently recruited from PrEP clients of a single internet hospital, HeHealth, in China. Although HeHealth is currently the largest online platform for obtaining PrEP in China, the use of a single-platform sample may limit the generalizability of the findings to broader populations of PrEP users. Second, both PrEP adherence monitoring data and survey data will rely on self-report, which may be subject to social desirability bias and recall bias and, therefore, may lead to overestimation of adherence. Third, loss to follow-up may pose a challenge because of the online recruitment strategy and the relatively long follow-up period. Although a 10% to 20% attrition rate has been considered in the sample size calculation, higher-than-expected dropout could still reduce the statistical power of the study. Fourth, the effectiveness of the intervention may vary according to the level of participant engagement with the platform. Because the intervention depends on users actively interacting with its functions, such as behavior recording, health education materials, medication reminders, expert consultation, and the chatbot, low engagement may weaken its effect on adherence and warrants further investigation into factors that promote or hinder sustained use. In addition, as behavioral monitoring itself may influence medication adherence through self-awareness or self-tracking effects, this trial can only estimate the incremental effect of the precision intervention functions beyond the monitoring function rather than the effect of the full digital platform compared with usual care. Finally, this study is limited to oral PrEP and does not include all PrEP dosage forms. With the introduction of long-acting injectable PrEP, adherence challenges may differ across modalities. However, oral PrEP is expected to remain the predominant form of PrEP use in China for the foreseeable future, and therefore, this study is focused specifically on oral PrEP users.

## Data Availability

No datasets were generated or analyzed in this study, as this is a study protocol. Data to be collected in the future study will not be publicly available due to the sensitive nature of the information, but deidentified data will be available from the corresponding author on reasonable request, subject to ethical approval.

## Appendix

Multimedia Appendix 1 Baseline questionnaire.

Multimedia Appendix 2 Follow-up questionnaire.

Multimedia Appendix 3 Peer review report by Young Scientists Fund Project Review Committee, National Natural Science Foundation of China.

Multimedia Appendix 4 SPIRIT Checklist.

## Abbreviations

AI: artificial intelligence
EMA: ecological momentary assessment
PrEP: preexposure prophylaxis
REMOTE: Real-Time Monitoring and Precision Intervention for HIV Preexposure Prophylaxis Adherence
SCT: stages of change theory
SPIRIT: Standard Protocol Items: Recommendations for Interventional Trials
TTM: transtheoretical model
