# Supplementary material for: Evaluation of a Digital Intervention for Monitoring and Improving Medication Adherence Among Real-World e-Consumers of HIV Preexposure Prophylaxis in China: Protocol for a Randomized Controlled Trial
Source: JMIR Res Protoc. 2026 Jun 29;15:e92750. doi: 10.2196/92750 (PMC13365895; doi:10.2196/92750)
Supplement: Multimedia Appendix 1 [file resprot_v15i1e92750_app1.pdf]

2025 HeHealth – Tsinghua University Health Service Survey English Translation

1. **What are your year and month of birth? __________ year __________ month**
   *[Scroll down] **
2. **What sex were you registered as at birth?**
   *[Single choice] **
   ○ Male
   ○ Female
3. **Where do you currently live?**
   *[Single choice] **
   ○ Urban area
   ○ Rural area
4. **What is your ethnicity?**
   *[Single choice] **
   ○ Han
   ○ Other ethnic group (please specify): __________
5. **What is your occupation?**
   *[Single choice] **
   ○ Student
   ○ Person in charge of a government agency, Party organization, enterprise, or public institution
   ○ Professional or technical personnel (for example, personnel in internet, finance, research, engineering, education, culture, or sports)
   ○ Clerical staff and related personnel (for example, personnel in administration, community services, security, or firefighting)
   ○ Commercial or service personnel
   ○ Agriculture, forestry, animal husbandry, fishery, or water conservancy production personnel
   ○ Production and transportation equipment operators and related personnel
   ○ Military personnel
   ○ Unemployed
   ○ Other occupation (please specify): __________
6. **What is the highest degree you have obtained, or are currently pursuing?**
   *[Single choice] **
   ○ Primary school or below
   ○ Junior high school
   ○ High school / vocational high school / technical secondary school
   ○ College / undergraduate
   ○ Master’s degree or above
7. **What is your current monthly income / average monthly living expenses (if you do not have income, report your average monthly living expenses), in RMB?**
   *[Single choice] **
   ○ Below RMB 3,000
   ○ RMB 3,001–5,000

○ RMB 5,001–7,000
○ RMB 7,001–10,000
○ RMB 10,001–15,000
○ RMB 15,001–20,000
○ Above RMB 20,000

8. **What is your current marital / relationship status?**
   [Single choice] *
   ○ Married or living with a partner
   ○ Unmarried
   ○ Divorced or living separately from a partner
   ○ Widowed

9. **Which of the following best describes your gender?**
   [Single choice] *
   ○ Male
   ○ Female
   ○ Transgender man (female-to-male)
   ○ Transgender woman (male-to-female)
   ○ Non-binary
   ○ Other

10. **Which PrEP regimen are you currently using?**
   [Single choice] *
   ○ Daily dosing regimen (please skip to Question 31)
   ○ Event-driven dosing regimen (also called on-demand dosing regimen)

11. **Which of the following are correct ways to take event-driven PrEP?**
   [Multiple choice] *
   □ First, take 2 pills orally 2–24 hours before anticipated sexual activity
   □ First, take 1 pill orally 2–24 hours before anticipated sexual activity
   □ After sexual activity, take 1 additional pill 24 hours after the first dose
   □ After sexual activity, take 1 additional pill 48 hours after the first dose
   □ If you miss one PrEP dose, you must take double the dose at the next dosing time
   □ If the interval between the last dose and the next sexual activity is <7 days, you should resume 1 pill daily until 48 hours after the last sexual activity
   □ Do not know / not sure

12. **In the past 3 months, have you had sexual intercourse (including oral, anal, or vaginal sex)?**
   [Single choice] *
   ○ Yes, I have had sexual intercourse
   ○ No, I have not had sexual intercourse (please skip to Question 37)

13. **In the past 3 months, how many times in total did you have sexual intercourse (including oral, anal, or vaginal sex)?**
   [Single choice] *
   ○ 1
   ○ 2
   ○ 3
   ○ 4

○ 5
○ 6
○ 7
○ 8
○ 9
○ 10
○ More than 10

14. **In the past 3 months, did you use condoms during every sexual encounter?**
[Single choice] *
○ Never
○ Occasionally
○ Sometimes
○ Often
○ Every time

15. **In the past 3 months, did you take PrEP medication during every sexual encounter?**
[Single choice] *
○ Yes, I took it during every sexual encounter (please skip to Question 17)
○ I took it only sometimes
○ No, I did not take it during any sexual encounter (please skip to Question 20)

16. **For how many sexual encounters did you not take PrEP?**
[Single choice] *
○ 1
○ 2
○ 3
○ 4
○ 5
○ 6
○ 7
○ 8
○ 9
○ 10
○ More than 10

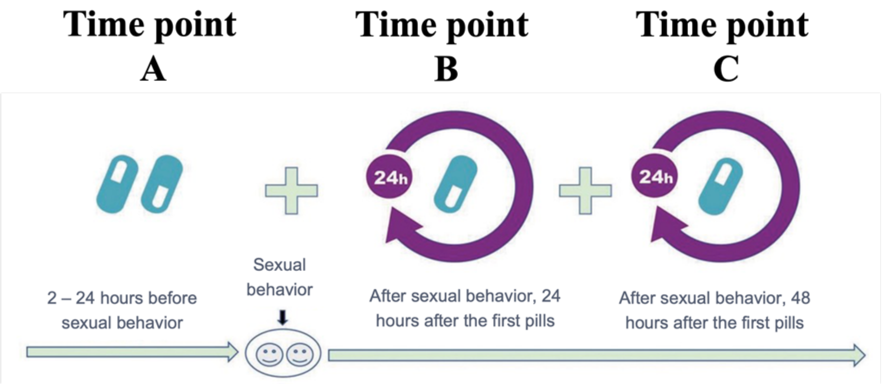

17. **In the past 3 months, when you used event-driven PrEP, did you take 2 PrEP pills 2–24 hours before the actual sexual activity (time point A)?**
[Single choice] *

○ Whenever I needed to take PrEP, I took 2 pills at time point A every time
○ Whenever I needed to take PrEP, I took 2 pills at time point A most of the time
○ Whenever I needed to take PrEP, I took 2 pills at time point A about half of the time
○ Whenever I needed to take PrEP, I took 2 pills at time point A sometimes
○ Whenever I needed to take PrEP, I never took 2 pills at time point A

18. **In the past 3 months, when you used event-driven PrEP, did you take 1 additional PrEP pill 24 hours after the first dose (time point B)?**

[Single choice] *

○ Whenever I needed to take PrEP, I took 1 pill at time point B every time
○ Whenever I needed to take PrEP, I took 1 pill at time point B most of the time
○ Whenever I needed to take PrEP, I took 1 pill at time point B about half of the time
○ Whenever I needed to take PrEP, I took 1 pill at time point B sometimes
○ Whenever I needed to take PrEP, I never took 1 pill at time point B

19. **In the past 3 months, when you used event-driven PrEP, did you take 1 additional PrEP pill 48 hours after the first dose (time point C)?**

[Single choice] *

○ Whenever I needed to take PrEP, I took 1 pill at time point C every time
○ Whenever I needed to take PrEP, I took 1 pill at time point C most of the time
○ Whenever I needed to take PrEP, I took 1 pill at time point C about half of the time
○ Whenever I needed to take PrEP, I took 1 pill at time point C sometimes
○ Whenever I needed to take PrEP, I never took 1 pill at time point C

20. **In the past 3 months, what were the reasons that you did not take the medication on time and at the prescribed dose (including situations in which sexual activity occurred and PrEP was not taken at all)?**

[Multiple choice] *

□ Not applicable; I took the medication on time and at the prescribed dose every time
□ I do not understand the correct way to take PrEP
□ Forgot to take the medication
□ Could not obtain the medication in time
□ The medication had side effects
□ I was worried that it would interfere with treatment for other illnesses
□ I was worried about privacy disclosure
□ People around me did not support it
□ It was inconvenient to take the medication
□ I used other protective measures (for example, condoms)
□ I trust my sexual partner
□ Other reason: ___________

21. **What side effects / adverse reactions have you experienced while taking PrEP?**

[Multiple choice] * *(Displayed only if Option 5 of Question 20 is selected.)*

□ Dizziness or headache
□ Nausea or vomiting
□ Abdominal pain or diarrhea
□ Liver or kidney injury
□ Fever
□ Fatigue / drowsiness
□ Insomnia / vivid dreams

□ Rash
□ Anxiety, depression, or other psychological abnormalities
□ Other side effect / adverse reaction: __________

22. **In the past 1 month, have you had sexual intercourse (including oral, anal, or vaginal sex)?**
[Single choice] *
○ Yes, I have had sexual intercourse
○ No, I have not had sexual intercourse (please skip to Question 37)

23. **In the past 1 month, how many times in total did you have sexual intercourse (including oral, anal, or vaginal sex)?**
[Single choice] *
○ 1
○ 2
○ 3
○ 4
○ 5
○ 6
○ 7
○ 8
○ 9
○ 10
○ More than 10

24. **In the past 1 month, did you use condoms during every sexual encounter?**
[Single choice] *
○ Never
○ Occasionally
○ Sometimes
○ Often
○ Every time

25. **In the past 1 month, did you take PrEP medication during every sexual encounter?**
[Single choice] *
○ Yes, I took it during every sexual encounter (please skip to Question 27)
○ I took it only sometimes
○ No, I did not take it during any sexual encounter (please skip to Question 30)

26. **For how many sexual encounters did you not take PrEP?**
[Single choice] *
○ 1
○ 2
○ 3
○ 4
○ 5
○ 6
○ 7
○ 8
○ 9
○ 10

○ More than 10

27. **In the past 1 month, when you used event-driven PrEP, did you take 2 PrEP pills 2–24 hours before the actual sexual activity (time point A)?**

    [Single choice] *

    ○ Whenever I needed to take PrEP, I took 2 pills at time point A every time
    ○ Whenever I needed to take PrEP, I took 2 pills at time point A most of the time
    ○ Whenever I needed to take PrEP, I took 2 pills at time point A about half of the time
    ○ Whenever I needed to take PrEP, I took 2 pills at time point A sometimes
    ○ Whenever I needed to take PrEP, I never took 2 pills at time point A

28. **In the past 1 month, when you used event-driven PrEP, did you take 1 additional PrEP pill 24 hours after the first dose (time point B)?**

    [Single choice] *

    ○ Whenever I needed to take PrEP, I took 1 pill at time point B every time
    ○ Whenever I needed to take PrEP, I took 1 pill at time point B most of the time
    ○ Whenever I needed to take PrEP, I took 1 pill at time point B about half of the time
    ○ Whenever I needed to take PrEP, I took 1 pill at time point B sometimes
    ○ Whenever I needed to take PrEP, I never took 1 pill at time point B

29. **In the past 1 month, when you used event-driven PrEP, did you take 1 additional PrEP pill 48 hours after the first dose (time point C)?**

    [Single choice] *

    ○ Whenever I needed to take PrEP, I took 1 pill at time point C every time
    ○ Whenever I needed to take PrEP, I took 1 pill at time point C most of the time
    ○ Whenever I needed to take PrEP, I took 1 pill at time point C about half of the time
    ○ Whenever I needed to take PrEP, I took 1 pill at time point C sometimes
    ○ Whenever I needed to take PrEP, I never took 1 pill at time point C

30. **In the past 1 month, what were the reasons that you did not take the medication on time and at the prescribed dose (including situations in which sexual activity occurred and PrEP was not taken at all)?**

    [Multiple choice] *

    □ Not applicable; I took the medication on time and at the prescribed dose every time
    □ I do not understand the correct way to take PrEP
    □ Forgot to take the medication
    □ Could not obtain the medication in time
    □ The medication had side effects
    □ I was worried that it would interfere with treatment for other illnesses
    □ I was worried about privacy disclosure
    □ People around me did not support it
    □ It was inconvenient to take the medication
    □ I used other protective measures (for example, condoms)
    □ I trust my sexual partner
    □ Other reason: ___________

[After completing this item, please skip to Question 37.]

31. **Which of the following are correct ways to take daily PrEP?**

[Multiple choice] *

- □ Take 1 pill every 24 hours
- □ Take 2 pills every 24 hours
- □ You must take it continuously for 7 days before engaging in HIV high-risk behavior
- □ You need to continue taking it for 7 days after the last HIV high-risk behavior before you can stop
- □ You can stop taking it immediately after the last HIV high-risk behavior
- □ If you miss one PrEP dose, you must take double the dose at the next dosing time
- □ Do not know / not sure

32. **In the past 1 month, have you had sexual intercourse (including oral, anal, or vaginal sex)?**

*[Single choice] **

- ○ Yes, I have had sexual intercourse
- ○ No, I have not had sexual intercourse (please skip to Question 34)

33. **In the past 1 month, how many times in total did you have sexual intercourse?**

*[Single choice] **

- ○ 1
- ○ 2
- ○ 3
- ○ 4
- ○ 5
- ○ 6
- ○ 7
- ○ 8
- ○ 9
- ○ 10
- ○ More than 10

34. **In the past 1 month, have you failed to take PrEP on time and at the prescribed dose?**

*[Single choice] **

- ○ Yes; the number of missed pills was __________
- ○ I did not miss any doses (please skip to Question 37)

35. **What were the reasons that you did not take the medication on time and at the prescribed dose?**

*[Multiple choice] **

- □ I do not understand the correct way to take PrEP
- □ Forgot to take the medication
- □ Could not obtain the medication in time
- □ The medication had side effects
- □ I was worried that it would interfere with treatment for other illnesses
- □ I was worried about privacy disclosure
- □ People around me did not support it
- □ It was inconvenient to take the medication
- □ I used other protective measures (for example, condoms)
- □ I trust my sexual partner
- □ I did not have sex very often
- □ Other reason: __________

36. **What side effects / adverse reactions have you experienced while taking PrEP?**

*[Multiple choice] **

- □ Dizziness or headache
- □ Nausea or vomiting
- □ Abdominal pain or diarrhea
- □ Liver or kidney injury
- □ Fever
- □ Fatigue / drowsiness
- □ Insomnia / vivid dreams
- □ Rash
- □ Anxiety, depression, or other psychological abnormalities
- □ Other side effect / adverse reaction: __________

Displayed only if Option 4 of Question 35 is selected.

37. **In the past 3 months, have you switched your PrEP dosing regimen?**

*[Multiple choice] **

- □ Yes, I switched from event-driven dosing to daily dosing
- □ Yes, I switched from daily dosing to event-driven dosing
- □ Yes, I switched to post-exposure prophylaxis (PEP)
- □ No

38. **Regarding taking PrEP consistently and maintaining good PrEP adherence, to what extent do you agree with the following statements?**

*[Matrix scale] **

Response options: Strongly disagree / Disagree / Neutral / Agree / Strongly agree

Item	Strongly disagree	Disagree	Neutral	Agree	Strongly agree
Only by maintaining good PrEP adherence can I avoid HIV infection	○	○	○	○	○
Only by maintaining good PrEP adherence can I protect people around me from HIV infection	○	○	○	○	○
I think continuing to take PrEP is too expensive	○	○	○	○	○
I am worried about the long-term side effects of PrEP	○	○	○	○	○
I worry that even if I maintain good	○	○	○	○	○

Item	Strongly disagree	Disagree	Neutral	Agree	Strongly agree
PrEP adherence, it still cannot completely prevent HIV					
My risk of acquiring HIV is low, so there is no need to maintain good PrEP adherence	○	○	○	○	○
Maintaining good PrEP adherence is somewhat difficult for me	○	○	○	○	○
Maintaining good PrEP adherence may lead me to have condomless sex	○	○	○	○	○
If I maintain good PrEP adherence, my partner may expect me to have condomless sex	○	○	○	○	○
This is an attention-check item. Please select Strongly agree	○	○	○	○	○
When I am currently drinking / have recently been drinking alcohol or using other drugs, I am still confident that I can continue taking PrEP	○	○	○	○	○
Even if my sexual partner is unwilling, I am still	○	○	○	○	○

Item	Strongly disagree	Disagree	Neutral	Agree	Strongly agree
confident that I can continue taking PrEP					
When I feel that it has side effects, I am still confident that I can continue taking PrEP	○	○	○	○	○
When using PrEP causes too much trouble for me, I am still confident that I can continue taking it	○	○	○	○	○
When I think my risk of acquiring HIV is low, I am still confident that I can continue taking PrEP	○	○	○	○	○
When I have already used other protective measures (for example, condoms), I am still confident that I can continue taking PrEP	○	○	○	○	○

39. **Regarding PrEP use, to what extent do you agree with the following statements?**
[Matrix scale] *

Response options: Strongly disagree / Disagree / Neutral / Agree / Strongly agree

Item	Strongly disagree	Disagree	Neutral	Agree	Strongly agree
I would feel ashamed to take PrEP in front of others	○	○	○	○	○
I think people who take PrEP should hide	○	○	○	○	○

Item	Strongly disagree	Disagree	Neutral	Agree	Strongly agree
their medication					
People who take PrEP will experience negative judgment	○	○	○	○	○
People who take PrEP are promiscuous	○	○	○	○	○
People who take PrEP are responsible	○	○	○	○	○
My friends support my PrEP use	○	○	○	○	○
I would encounter trouble if I told my sexual partner that I am taking PrEP	○	○	○	○	○
I would feel proud to take PrEP	○	○	○	○	○
People who take PrEP will experience verbal harassment	○	○	○	○	○
People who take PrEP are taking responsibility for their own health	○	○	○	○	○
My family would support my PrEP use	○	○	○	○	○

40. **Do you use, or do you plan to use, any tools or methods to remind yourself to take PrEP (for example, a phone alarm, an electronic pillbox, or reminders from relatives or friends)?**
    [Single choice] *
    ○ Yes: __________
    ○ No

41. **In the past 3 months, have you had sex with same-sex partners (anal or oral sex)?**
    [Single choice] *
    ○ Yes, I have had ______ same-sex partner(s) in the past 3 months (enter a number; minimum value = 1): __________

○ No (please skip to Question 49)

42. **In the past 3 months, have you had sex with a regular same-sex partner (anal or oral sex)?**
[Single choice] *
○ Yes, I have a regular sexual partner and we had sex
○ No (please skip to Question 45)

43. **In the past 3 months, when you had sex with your regular same-sex partner, what was your sexual role?**
[Single choice] *
○ Exclusively insertive (pure top)
○ Mainly insertive (mainly top)
○ Both, about equally
○ Mainly receptive (mainly bottom)
○ Exclusively receptive (pure bottom)

44. **In the past 3 months, how often did you use condoms when having sex with your regular same-sex partner?**
[Single choice] *
○ Never
○ Occasionally
○ Sometimes
○ Often
○ Every time

45. **In the past 3 months, have you had sex with casual same-sex partners (anal or oral sex)?**
[Single choice] *
○ Yes, I have had ______ casual same-sex partner(s) in the past 3 months (enter a number; minimum value = 1): __________
○ No (please skip to Question 48)

46. **In the past 3 months, when you had sex with your casual same-sex partners, what was your sexual role?**
[Single choice] *
○ Exclusively insertive (pure 1)
○ Mainly insertive (mainly 1)
○ Both, about equally
○ Mainly receptive (mainly 0)
○ Exclusively receptive (pure 0)

47. **In the past 3 months, how often did you use condoms when having sex with your casual same-sex partners?**
[Single choice] *
○ Never
○ Occasionally
○ Sometimes
○ Often
○ Every time

48. **In the past 3 months, did you use any of the following substances when engaging in same-sex sexual activity?**

*[Multiple choice] **

◻ Hallucinogens (for example, zero capsules, ecstasy, psychedelic mushrooms, trips, ketamine, and so on)
◻ Inhalants (for example, rush, poppers, nitrites, glue sniffing, solvent sniffing, and so on)
◻ Cannabis products (for example, marijuana, cannabis buds, cannabis herb, hashish, and so on)
◻ Amphetamine-type stimulants (for example, amphetamine pills, methamphetamine, ecstasy, and so on)
◻ I did not use any of the above substances

49. **In the past 3 months, have you had sex with opposite-sex partners (anal, vaginal, or oral sex)?**

*[Single choice] **

○ Yes, I have had ______ partner(s) in total (enter a number; minimum value = 1, including both regular and casual partners): __________
○ No (please skip to Question 51)

50. **In the past 3 months, how often did you use condoms when having sex with opposite-sex partners?**

*[Single choice] **

○ Never
○ Occasionally
○ Sometimes
○ Often
○ Every time

51. **Do you undergo regular HIV testing?**

*[Single choice] **

○ Yes, I undergo regular testing, and my testing frequency is once every _____ months: __________
○ Yes, I have had HIV testing, but not regularly
○ No, I have never had HIV testing (please skip to Question 54)

52. **In the past 3 months, have you had an HIV test?**

*[Single choice] **

○ Yes
○ No

53. **What was the result of your most recent HIV test?**

*[Single choice] **

○ Negative
○ Positive
○ Uncertain / do not know

54. **In the past 3 months, have you had any of the following diseases?**

*[Multiple choice] **

◻ Syphilis
◻ Gonorrhea
◻ Genital warts
◻ Genital herpes
◻ Genital Chlamydia trachomatis infection

- ◻ Hepatitis B
- ◻ Hepatitis C
- ◻ Mpox
- ◻ Other sexually transmitted disease, please specify: __________
- ◻ Unclear / do not know
- ◻ None of the above

55. **If you maintain good PrEP adherence, how likely do you think it is that you will acquire HIV in the next year?**

    [Single choice] *

    - ○ Very unlikely
    - ○ Unlikely
    - ○ Moderate
    - ○ Likely
    - ○ Very likely

56. **If you cannot maintain good PrEP adherence, how likely do you think it is that you will acquire HIV in the next year?**

    [Single choice] *

    - ○ Very unlikely
    - ○ Unlikely
    - ○ Moderate
    - ○ Likely
    - ○ Very likely

57. **Overall, how likely do you think it is that you will acquire HIV in the next year?**

    [Single choice] *

    - ○ Very unlikely
    - ○ Unlikely
    - ○ Moderate
    - ○ Likely
    - ○ Very likely

58. **How much impact do you think HIV infection would have on a person's health?**

    [Single choice] *

    - ○ Very little
    - ○ Little
    - ○ Moderate
    - ○ Great
    - ○ Very great

59. **How much impact do you think HIV infection would have on a person's life?**

    [Single choice] *

    - ○ Very little
    - ○ Little
    - ○ Moderate
    - ○ Great
    - ○ Very great

60. **Over the past two weeks, how often have you been bothered by any of the following problems?**
*[Matrix scale] **

Response options: Not at all / Several days / More than half the days / Nearly every day

Item	Not at all	Several days	More than half the days	Nearly every day
Little interest or pleasure in doing things	○	○	○	○
Feeling down, depressed, or hopeless	○	○	○	○
Trouble falling asleep, staying asleep, or sleeping too much	○	○	○	○
This is an attention-check item. Please select Nearly every day	○	○	○	○
Feeling tired or having little energy	○	○	○	○
Poor appetite or overeating	○	○	○	○
Feeling bad about yourself — or feeling that you are a failure or have let yourself or your family down	○	○	○	○
Trouble concentrating on things, such as reading the newspaper or watching television	○	○	○	○
Moving or speaking so slowly that other people could have noticed, or the opposite — being so fidgety or restless that you have been moving around more than usual	○	○	○	○
Thoughts that you would be better off	○	○	○	○

Item	Not at all	Several days	More than half the days	Nearly every day
dead, or thoughts of hurting yourself in some way				

61. **The following questions ask how you try to cope when you face difficulties in life. Please read the statements below and select the option that best reflects how often you use each coping method.**
    *[Matrix scale] **

Response options: I have never done this / I do this occasionally / I do this about half the time / I always do this

Item	Never do this	Occasionally do this	Do this about half the time	Always do this
I concentrate my efforts on dealing with the difficulties I am facing	○	○	○	○
I get emotional support from others	○	○	○	○
I give up trying to deal with the difficulties	○	○	○	○
I take practical action to improve the situation	○	○	○	○
I get help and advice from others	○	○	○	○
I try to think of strategies to solve the difficulties	○	○	○	○
I get comfort and understanding from others	○	○	○	○
I give up trying to cope with the difficulties	○	○	○	○
I seek help or advice from others about how to cope with the difficulties	○	○	○	○
I think hard about what steps to take next to solve the difficulties	○	○	○	○

62. **Based on your situation over the past month, please select the option that best describes you for each of the following statements.**
    *[Matrix scale] **

Response options: Never / Rarely / Sometimes / Often / Always

Item	Never	Rarely	Sometimes	Often	Always
I am able to adapt to change	○	○	○	○	○
I tend to bounce back quickly after hardship or illness	○	○	○	○	○

63. **Over the past two weeks, how often have you been bothered by the following problems?** *[Matrix scale] **

Response options: Not at all / Several days / More than half the days / Nearly every day

Item	Not at all	Several days	More than half the days	Nearly every day
Feeling nervous, anxious, or on edge	○	○	○	○
Not being able to stop or control worrying	○	○	○	○
Worrying too much about different things	○	○	○	○
This is an attention-check item. Please select Nearly every day	○	○	○	○
Trouble relaxing	○	○	○	○
Being so restless that it is hard to sit still	○	○	○	○
Becoming easily annoyed or irritable	○	○	○	○
Feeling afraid as if something awful might happen	○	○	○	○
