# Supplementary material for: Evaluation of a Digital Intervention for Monitoring and Improving Medication Adherence Among Real-World e-Consumers of HIV Preexposure Prophylaxis in China: Protocol for a Randomized Controlled Trial
Source: JMIR Res Protoc. 2026 Jun 29;15:e92750. doi: 10.2196/92750 (PMC13365895; doi:10.2196/92750)
Supplement: Multimedia Appendix 2 [file resprot_v15i1e92750_app2.pdf]

2026 HeHealth – Tsinghua University Health Service Survey

1-Month Follow-up Questionnaire (English Translation)

1. What is your date of birth? *[Scroll down]*

______ Year ______ Month

2. What is your current marital or relationship status? *[Single choice]*

○ Married or living with a partner
○ Unmarried
○ Divorced or separated from partner
○ Widowed

3. Which PrEP regimen are you currently using? *[Single choice]*

○ Daily PrEP regimen (skip to Question 23)
○ Event-driven PrEP regimen (also called on-demand PrEP)

4. Which of the following describe the correct way to take event-driven PrEP? *[Multiple choice]*

□ Take 2 pills orally 2 to 24 hours before anticipated sexual activity
□ Take 1 pill orally 2 to 24 hours before anticipated sexual activity
□ Take 1 additional pill 24 hours after the first dose
□ Take 1 additional pill 48 hours after the first dose
□ If you miss one PrEP dose, you must take double the dose at the next dosing time
□ If the interval between the last dose and the next sexual activity is <7 days, you should resume 1 pill daily until 48 hours after the last sexual activity
□ Do not know / not sure

5. In the past 3 months, have you had sexual intercourse (including oral, anal, or vaginal sex)? *[Single choice]*

○ Yes, I have had sexual intercourse
○ No, I have not had sexual intercourse (skip to Question 29)

6. In the past 3 months, how many times did you have sexual intercourse (including oral, anal, or vaginal sex)? *[Single choice]*

○ 1
○ 2
○ 3
○ 4
○ 5
○ 6
○ 7
○ 8
○ 9
○ 10
○ More than 10

7. In the past 3 months, did you use condoms during every sexual intercourse? *[Single choice]*

○ Never
○ Rarely

○ Sometimes
○ Often
○ Every time

8. In the past 3 months, did you take PrEP during every sexual intercourse? *[Single choice]*

○ Yes, I took it every time (skip to Question 10)
○ Only sometimes
○ I did not take it at any sexual intercourse (skip to Question 13)

9. In how many sexual encounters did you not take PrEP? *[Single choice]*

○ 1
○ 2
○ 3
○ 4
○ 5
○ 6
○ 7
○ 8
○ 9
○ 10
○ More than 10

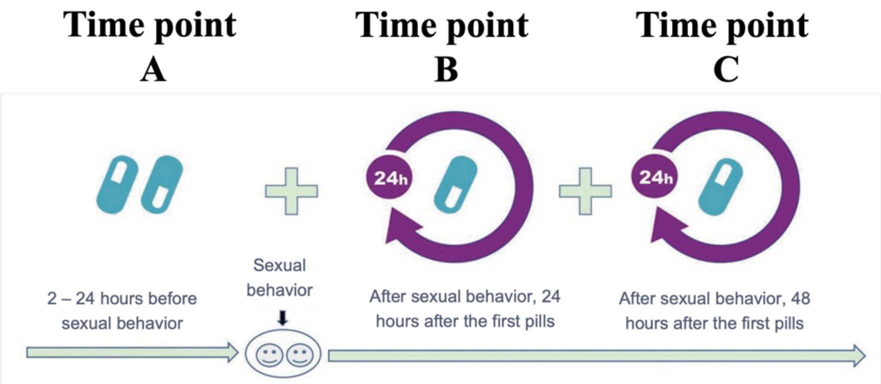

10. In the past 3 months, when you used event-driven PrEP, did you take 2 PrEP pills 2 to 24 hours before the actual sexual activity (Time Point A)? *[Single choice]*

○ Whenever I needed to take PrEP, I took 2 pills at Time Point A every time
○ Whenever I needed to take PrEP, I took 2 pills at Time Point A most of the time
○ Whenever I needed to take PrEP, I took 2 pills at Time Point A about half of the time
○ Whenever I needed to take PrEP, I took 2 pills at Time Point A sometimes
○ Whenever I needed to take PrEP, I never took 2 pills at Time Point A

11. In the past 3 months, when you used event-driven PrEP, did you take 1 additional PrEP pill 24 hours after the first dose (Time Point B)? *[Single choice]*

○ Whenever I needed to take PrEP, I took 1 pill at Time Point B every time
○ Whenever I needed to take PrEP, I took 1 pill at Time Point B most of the time
○ Whenever I needed to take PrEP, I took 1 pill at Time Point B about half of the time
○ Whenever I needed to take PrEP, I took 1 pill at Time Point B sometimes
○ Whenever I needed to take PrEP, I never took 1 pill at Time Point B

12. In the past 3 months, when you used event-driven PrEP, did you take 1 additional PrEP pill 48 hours after the first dose (Time Point C)? *[Single choice]*

○ Whenever I needed to take PrEP, I took 1 pill at Time Point C every time
○ Whenever I needed to take PrEP, I took 1 pill at Time Point C most of the time
○ Whenever I needed to take PrEP, I took 1 pill at Time Point C about half of the time
○ Whenever I needed to take PrEP, I took 1 pill at Time Point C sometimes
○ Whenever I needed to take PrEP, I never took 1 pill at Time Point C

13. In the past 3 months, what were the reasons that you did not take PrEP on time and at the prescribed dose (including having sex and not taking any PrEP at all)? *[Multiple choice]*

□ Not applicable, I took the medication on time and at the prescribed dose every time
□ I do not understand the correct way to take PrEP
□ Forgot to take the medication
□ Could not obtain the medication in time
□ The medication had side effects
□ I was concerned that it might interfere with treatment for other diseases
□ I was concerned about privacy disclosure
□ People around me did not support it
□ Taking medication was inconvenient
□ I used other protective measures (e.g., condoms)
□ I trust my sexual partner
□ Other reason: ______

14. In the past 1 month, have you had sexual intercourse (including oral, anal, or vaginal sex)? *[Single choice]*

○ Yes, I have had sexual intercourse
○ No, I have not had sexual intercourse (skip to Question 29)

15. In the past 1 month, how many times did you have sexual intercourse (including oral, anal, or vaginal sex)? *[Single choice]*

○ 1
○ 2
○ 3
○ 4
○ 5
○ 6
○ 7
○ 8
○ 9
○ 10
○ More than 10

16. In the past 1 month, did you use condoms during every sexual intercourse? *[Single choice]*

○ Never
○ Rarely
○ Sometimes
○ Often
○ Every time

17. In the past 1 month, did you take PrEP during every sexual intercourse? *[Single choice]*

○ Yes, I took it every time (skip to Question 19)
○ Only sometimes
○ I did not take it at any sexual intercourse (skip to Question 22)

18. In how many sexual encounters did you not take PrEP? *[Single choice]*

○ 1
○ 2
○ 3
○ 4
○ 5
○ 6
○ 7
○ 8
○ 9
○ 10
○ More than 10

19. In the past 1 month, when you used event-driven PrEP, did you take 2 PrEP pills 2 to 24 hours before the actual sexual activity (Time Point A)? *[Single choice]*

○ Whenever I needed to take PrEP, I took 2 pills at Time Point A every time
○ Whenever I needed to take PrEP, I took 2 pills at Time Point A most of the time
○ Whenever I needed to take PrEP, I took 2 pills at Time Point A about half of the time
○ Whenever I needed to take PrEP, I took 2 pills at Time Point A sometimes
○ Whenever I needed to take PrEP, I never took 2 pills at Time Point A

20. In the past 1 month, when you used event-driven PrEP, did you take 1 additional PrEP pill 24 hours after the first dose (Time Point B)? *[Single choice]*

○ Whenever I needed to take PrEP, I took 1 pill at Time Point B every time
○ Whenever I needed to take PrEP, I took 1 pill at Time Point B most of the time
○ Whenever I needed to take PrEP, I took 1 pill at Time Point B about half of the time
○ Whenever I needed to take PrEP, I took 1 pill at Time Point B sometimes
○ Whenever I needed to take PrEP, I never took 1 pill at Time Point B

21. In the past 1 month, when you used event-driven PrEP, did you take 1 additional PrEP pill 48 hours after the first dose (Time Point C)? *[Single choice]*

○ Whenever I needed to take PrEP, I took 1 pill at Time Point C every time
○ Whenever I needed to take PrEP, I took 1 pill at Time Point C most of the time
○ Whenever I needed to take PrEP, I took 1 pill at Time Point C about half of the time
○ Whenever I needed to take PrEP, I took 1 pill at Time Point C sometimes
○ Whenever I needed to take PrEP, I never took 1 pill at Time Point C

22. In the past 1 month, what were the reasons that you did not take PrEP on time and at the prescribed dose (including having sex and not taking any PrEP at all)? *[Multiple choice]*

□ Not applicable, I took the medication on time and at the prescribed dose every time
□ I do not understand the correct way to take PrEP
□ Forgot to take the medication
□ Could not obtain the medication in time

□ The medication had side effects
□ I was concerned that it might interfere with treatment for other diseases
□ I was concerned about privacy disclosure
□ People around me did not support it
□ Taking medication was inconvenient
□ I used other protective measures (e.g., condoms)
□ I trust my sexual partner
□ Other reason: ______

After completing this question, please skip to Question 29.

23. Which of the following describe the correct way to take daily PrEP? *[Multiple choice]*

□ Take 1 pill every 24 hours
□ Take 2 pills every 24 hours
□ You must take it continuously for 7 days before engaging in high-risk HIV exposure
□ You need to continue taking it for 7 days after the last high-risk HIV exposure before stopping
□ You may stop immediately after the last high-risk HIV exposure
□ If you miss one PrEP dose, you must take double the dose at the next dosing time
□ Do not know / not sure

24. In the past 1 month, have you had sexual intercourse (including oral, anal, or vaginal sex)? *[Single choice]*

○ Yes, I have had sexual intercourse
○ No, I have not had sexual intercourse (skip to Question 26)

25. In the past 1 month, how many times did you have sexual intercourse? *[Single choice]*

○ 1
○ 2
○ 3
○ 4
○ 5
○ 6
○ 7
○ 8
○ 9
○ 10
○ More than 10

26. In the past 1 month, have you ever failed to take PrEP on time and at the prescribed dose? *[Single choice]*

○ Yes, the number of missed pills was: ______
○ I did not miss any doses (skip to Question 29)

27. What were the reasons that you did not take the medication on time and at the prescribed dose? *[Multiple choice]*

□ I do not understand the correct way to take PrEP
□ Forgot to take the medication
□ Could not obtain the medication in time
□ The medication had side effects
□ I was concerned that it might interfere with treatment for other diseases

- □ I was concerned about privacy disclosure
- □ People around me did not support it
- □ Taking medication was inconvenient
- □ I used other protective measures (e.g., condoms)
- □ I trust my sexual partner
- □ I did not have sex very often
- □ Other reason: ______

28. What side effects or adverse reactions have you experienced while taking PrEP? *[Multiple choice]*

- □ Dizziness or headache
- □ Nausea or vomiting
- □ Abdominal pain or diarrhea
- □ Liver or kidney impairment
- □ Fever
- □ Fatigue or drowsiness
- □ Insomnia or vivid dreams
- □ Skin rash
- □ Psychological symptoms such as anxiety or depression
- □ Other side effect / adverse reaction: ______

This item is dependent on the 4th option of Question 27.

29. In the past 3 months, have you changed your PrEP regimen? *[Multiple choice]*

- □ Yes, I changed from event-driven PrEP to daily PrEP
- □ Yes, I changed from daily PrEP to event-driven PrEP
- □ Yes, I switched to post-exposure prophylaxis (PEP)
- □ No

30. Regarding continued PrEP use and maintaining good PrEP adherence, to what extent do you agree with the following statements? *[Matrix scale]*

Statement	Strongly disagree	Disagree	Neutral	Agree	Strongly agree
Only by maintaining good PrEP adherence can I avoid HIV infection	○	○	○	○	○
Only by maintaining good PrEP adherence can I protect people around me from HIV infection	○	○	○	○	○
I think persisting with PrEP is too expensive	○	○	○	○	○

Statement	Strongly disagree	Disagree	Neutral	Agree	Strongly agree
I worry about the long-term side effects of PrEP	○	○	○	○	○
I worry that even if I maintain good PrEP adherence, it still may not completely prevent HIV	○	○	○	○	○
My risk of acquiring HIV is low, so there is no need to maintain good PrEP adherence	○	○	○	○	○
Maintaining good PrEP adherence is somewhat difficult for me	○	○	○	○	○
Maintaining good PrEP adherence may lead me to have condomless sex	○	○	○	○	○
If I maintain good PrEP adherence, my partner may expect me to have condomless sex	○	○	○	○	○
This is an attention check item. Please select 'Strongly agree'	○	○	○	○	○
When I am currently using or have recently used alcohol or other drugs, I	○	○	○	○	○

Statement	Strongly disagree	Disagree	Neutral	Agree	Strongly agree
still feel confident that I can continue taking PrEP					
Even if my sexual partner is unwilling, I still feel confident that I can continue taking PrEP	○	○	○	○	○
When I feel that it has side effects, I still feel confident that I can continue taking PrEP	○	○	○	○	○
When using PrEP causes me too much trouble, I still feel confident that I can continue taking it	○	○	○	○	○
When I think my risk of acquiring HIV is low, I still feel confident that I can continue taking PrEP	○	○	○	○	○
When I have already used other protective measures (e.g., condoms), I still feel confident that I can continue taking PrEP	○	○	○	○	○

31. Regarding PrEP use, to what extent do you agree with the following statements? *[Matrix scale]*

Statement	Strongly disagree	Disagree	Neutral	Agree	Strongly agree
I would feel ashamed to take PrEP in front of other people	○	○	○	○	○
I think people who take PrEP should hide the medication	○	○	○	○	○
People who take PrEP experience negative judgments	○	○	○	○	○
People who take PrEP are promiscuous	○	○	○	○	○
People who take PrEP are responsible	○	○	○	○	○
My friends support my taking PrEP	○	○	○	○	○
I would have trouble telling my sexual partner that I am taking PrEP	○	○	○	○	○
I would feel proud to take PrEP	○	○	○	○	○
People who take PrEP are verbally harassed	○	○	○	○	○
People who take PrEP are taking responsibility for their health	○	○	○	○	○
My family would support my taking PrEP	○	○	○	○	○

32. Do you use, or do you plan to use, any tools or methods to remind yourself to take PrEP (for example, a mobile phone alarm, an electronic pillbox, or reminders from family or friends)? *[Single choice]*

○ Yes: ______
○ No

33. In the past 3 months, have you had sexual intercourse with men (anal or oral sex)? *[Single choice]*

○ Yes, I had sexual intercourse with ______ male partner(s) in the past 3 months (enter a number, minimum value = 1): ______
○ No (skip to Question 41)

34. In the past 3 months, have you had sexual intercourse (anal or oral sex) with a steady male partner (for example, a boyfriend, or someone with whom you have a long-term or repeated sexual relationship)? *[Single choice]*

○ Yes, I had a steady partner and had sex
○ No (skip to Question 37)

35. In the past 3 months, what was your sexual role when having sex with your steady male partner (for example, a boyfriend, or someone with whom you have a long-term or repeated sexual relationship)? *[Single choice]*

○ Exclusively insertive (pure top, 1)
○ Mainly insertive (mostly top, mainly 1)
○ Both, about equally
○ Mainly receptive (mostly bottom, mainly 0)
○ Exclusively receptive (pure bottom, 0)

36. In the past 3 months, how often did you use condoms when having sex with your steady male partner (for example, a boyfriend, or someone with whom you have a long-term or repeated sexual relationship)? *[Single choice]*

○ Never
○ Rarely
○ Sometimes
○ Often
○ Every time

37. In the past 3 months, have you had sexual intercourse (anal or oral sex) with a casual male partner (for example, a sexual partner with whom you had sex only once or had an unstable relationship, and whom you met through apps, bars, friends, or other channels)? *[Single choice]*

○ Yes, I had ______ casual male partner(s) in the past 3 months (enter a number, minimum value = 1): ______
○ No (skip to Question 40)

38. In the past 3 months, what was your sexual role when having sex with a casual male partner (for example, a sexual partner with whom you had sex only once or had an unstable relationship, and whom you met through apps, bars, friends, or other channels)? *[Single choice]*

○ Exclusively insertive (pure top)
○ Mainly insertive (mostly top)
○ Both, about equally

○ Mainly receptive (mostly bottom)
○ Exclusively receptive (pure bottom)

39. In the past 3 months, how often did you use condoms when having sex with a casual male partner (for example, a sexual partner with whom you had sex only once or had an unstable relationship, and whom you met through apps, bars, friends, or other channels)? *[Single choice]*

○ Never
○ Rarely
○ Sometimes
○ Often
○ Every time

40. In the past 3 months, have you used any of the following substances when having sex with men? *[Multiple choice]*

□ Hallucinogens (for example, capsule No. 0, ecstasy, psychedelic mushrooms, trips, ketamine, etc.)
□ Inhalants (for example, rush, poppers, nitrites, glue sniffing, inhaling volatile oils, etc.)
□ Cannabis products (for example, marijuana, cannabis buds, cannabis herb, hashish, etc.)
□ Amphetamine-type stimulants (for example, yaba, methamphetamine, ecstasy, etc.)
□ I did not use any of the above substances

41. In the past 3 months, have you had sexual intercourse with women (anal, vaginal, or oral sex)? *[Single choice]*

○ Yes, I had a total of ______ female sexual partner(s) (enter a number, minimum value = 1, including both steady and casual partners): ______
○ No (skip to Question 43)

42. In the past 3 months, how often did you use condoms when having sex with women? *[Single choice]*

○ Never
○ Rarely
○ Sometimes
○ Often
○ Every time

43. Do you undergo regular HIV testing? *[Single choice]*

○ Yes, I undergo regular testing. My testing frequency is once every ______ months: ______
○ Yes, I have had HIV testing, but not regularly
○ No, I have never had HIV testing (skip to Question 46)

44. In the past 3 months, have you had an HIV test? *[Single choice]*

○ Yes
○ No

45. What was the result of your most recent HIV test? *[Single choice]*

○ Negative
○ Positive
○ Uncertain or do not know

46. In the past 3 months, have you ever had any of the following diseases? *[Multiple choice]*

□ Syphilis

◻ Gonorrhea
◻ Genital warts
◻ Genital herpes
◻ Genital chlamydia infection
◻ Hepatitis B
◻ Hepatitis C
◻ Mpox
◻ Other sexually transmitted disease, please specify: ______
◻ Unclear / do not know
◻ None of the above

47. If you maintain good PrEP adherence, what do you think is your likelihood of acquiring HIV in the next year? *[Single choice]*

○ Very low
○ Low
○ Moderate
○ High
○ Very high

48. If you cannot maintain good PrEP adherence, what do you think is your likelihood of acquiring HIV in the next year? *[Single choice]*

○ Very low
○ Low
○ Moderate
○ High
○ Very high

49. Overall, what do you think is your likelihood of acquiring HIV in the next year? *[Single choice]*

○ Very low
○ Low
○ Moderate
○ High
○ Very high

50. How much impact do you think acquiring HIV would have on a person's health? *[Single choice]*

○ Very low
○ Low
○ Moderate
○ High
○ Very high

51. How much impact do you think acquiring HIV would have on a person's life? *[Single choice]*

○ Very low
○ Low
○ Moderate
○ High
○ Very high

52. [Intervention group version] Over the past month, you used the PLP Camp platform for comprehensive health management (including health education, intelligent consultation, medication reminders, circle/forum functions, and other features). We would like to know your real experience. As a comprehensive digital health assistant, do you think it is easy to use? Has this system truly fit into your daily life? Please consider your experience across the platform's functions and indicate your views. *[Matrix scale]*

Statement	Strongly disagree	Disagree	Uncertain	Agree	Strongly agree
I support using the PLP platform to assist my PrEP use	○	○	○	○	○
The PLP platform's functions (such as check-in and health education) are attractive to me	○	○	○	○	○
I like using the PLP platform	○	○	○	○	○
I welcome this type of digital platform-based health management intervention	○	○	○	○	○
The PLP platform seems to fit my health management needs well	○	○	○	○	○
The PLP platform is well suited to help people like me continue taking PrEP	○	○	○	○	○
The PLP platform's functions fit my daily life habits	○	○	○	○	○
The PLP platform works well with my current PrEP plan	○	○	○	○	○

Statement	Strongly disagree	Disagree	Uncertain	Agree	Strongly agree
It is entirely feasible to use the PLP platform in my daily life	○	○	○	○	○
It is possible for me to continue using the PLP platform every day / every week	○	○	○	○	○
It is easy for me to complete tasks on the PLP platform (such as medication check-ins and reading health education materials)	○	○	○	○	○
I think the PLP platform is simple and convenient to operate	○	○	○	○	○

52. [Control group version] Over the past month, you used the PLP Camp platform check in online for medication adherence monitoring. We would like to know your real experience. As a comprehensive digital health assistant, do you think it is easy to use? Has this system truly fit into your daily life? Please consider your experience across the platform's functions and indicate your views. *[Matrix scale]*

Statement	Strongly disagree	Disagree	Uncertain	Agree	Strongly agree
I support using the PLP platform to record my PrEP use	○	○	○	○	○
This method of recording medication is appealing to me	○	○	○	○	○
I like using the PLP platform for check-ins	○	○	○	○	○

Statement	Strongly disagree	Disagree	Uncertain	Agree	Strongly agree
I welcome this self-management approach of using a mobile device to record medication intake	○	○	○	○	○
This check-in tool seems well-suited to my daily PrEP routine	○	○	○	○	○
This recording method is appropriate for helping someone like me who takes PrEP	○	○	○	○	○
The PLP platform's functions fit my daily life habits	○	○	○	○	○
This tool works well with my current PrEP medication schedule	○	○	○	○	○
Using this check-in tool to record PrEP intake in my daily life is entirely feasible	○	○	○	○	○
It is possible to consistently use this check-in tool to record PrEP intake	○	○	○	○	○
Completing the check-in to record PrEP intake is very easy for me	○	○	○	○	○
I think the check-in page of PLP platform	○	○	○	○	○

Statement	Strongly disagree	Disagree	Uncertain	Agree	Strongly agree
is simple and convenient to operate					

53. This section aims to understand your experience with the specific functional modules in the PLP Camp platform. Based on your actual use, please select the option that best matches your situation. *[Matrix scale, intervention group only]*

Function module	I do not know what this function is	Hardly ever used	Used sometimes	Used occasionally	Used often	Used all the time
Health education	○	○	○	○	○	○
Intelligent consultation	○	○	○	○	○	○
His/Her Story	○	○	○	○	○	○
Circle / forum	○	○	○	○	○	○
Intelligent medication reminders (not check-ins)	○	○	○	○	○	○
Expert consultation	○	○	○	○	○	○

54. During this month of use, what were the greatest facilitating factors or advantages of the platform for you? *[Multiple choice, intervention group only]*

□ The platform's medication reminder function helps me take medication on time
□ The health education content provided by the platform is practically helpful to me
□ The platform updates information promptly, and the content is clearly presented and easy to understand
□ The platform's overall operating process is relatively simple and convenient to use
□ The content pushed by the platform matches my personal situation relatively well
□ The platform overall gives a professional and reliable impression
□ Using the platform gives me more confidence in managing my health
□ The platform makes me pay more attention to my PrEP use and health status
□ The platform's rich functions increase my willingness to continue using it
□ When using the platform, I feel that someone is supporting and reminding me
□ I hope to use the platform to better manage my PrEP use and follow-up care

□ Other (please specify: ______)

55. During this month of use, what were the greatest barriers or difficulties you encountered when using the platform? *[Multiple choice, intervention group only]*

□ The platform has many functions, and I am not sure which ones I should use first
□ Some functions involve too many steps and are not convenient enough to use
□ The timing of the platform's pushed messages or medication reminders is not very suitable
□ The content provided by the platform does not fully match my current practical needs
□ I occasionally encountered technical problems during use (such as slow loading, lagging, or failure to open)
□ I am not very clear about what some functions do or how to use them
□ My daily life or work is busy, so I easily forget to open or use the platform
□ Using the platform requires extra time and is somewhat burdensome for me
□ I have some concerns about personal privacy or information security
□ I lack motivation to continue using the platform over the long term
□ I am not sure whether the platform can really help me improve medication use or health management
□ Other (please specify: ______)

Under the following five headings, please choose the statement that best describes your health today.

56. Mobility *[Single choice]*

○ I have no problems in walking about
○ I have slight problems in walking about
○ I have moderate problems in walking about
○ I have severe problems in walking about
○ I am unable to walk about

57. Self-care *[Single choice]*

○ I have no problems washing or dressing myself
○ I have slight problems washing or dressing myself
○ I have moderate problems washing or dressing myself
○ I have severe problems washing or dressing myself
○ I am unable to wash or dress myself

58. Usual activities (for example, work, study, housework, family, or leisure activities) *[Single choice]*

○ I have no problems doing my usual activities
○ I have slight problems doing my usual activities
○ I have moderate problems doing my usual activities
○ I have severe problems doing my usual activities
○ I am unable to do my usual activities

59. Pain or discomfort *[Single choice]*

○ I have no pain or discomfort
○ I have slight pain or discomfort
○ I have moderate pain or discomfort
○ I have severe pain or discomfort
○ I have extreme pain or discomfort

60. Anxiety or depression *[Single choice]*

○ I am not anxious or depressed
○ I am slightly anxious or depressed
○ I am moderately anxious or depressed
○ I am severely anxious or depressed
○ I am extremely anxious or depressed

61. We would like to know how good or bad your health is today. On the scale below, 100 represents the best health you can imagine and 0 represents the worst health you can imagine. [Enter a number from 0 to 100] *[Open-ended]*

____

62. In the past 3 months, how much in total did you spend on purchasing PrEP medication, in RMB? *[Open-ended]*

____

63. In the past 3 months, how many times did you go to a hospital or a Centers for Disease Control and Prevention (CDC) clinic for PrEP-related physical examinations (such as HIV testing, renal function tests, or sexually transmitted infection screening)? *[Single choice]*

○ Did not go
○ 1 time
○ 2 times
○ 3 times
○ 4 times
○ 5 times or more

64. Across these visits, how much did you pay in total (including registration fees, examination fees, and materials fees)? *[Open-ended]*

____

This item is dependent on options 2, 3, 4, 5, or 6 of Question 63.

65. Apart from spending at medical institutions, in the past 3 months, did you purchase any self-testing kits on your own (such as HIV urine/blood self-test kits or syphilis test strips)? *[Single choice]*

○ No, I did not purchase any
○ Yes, I did purchase some

66. How much in total did you spend on purchasing these self-testing kits? *[Open-ended]*

____

This item is dependent on option 2 of Question 65.

67. Over the past 2 weeks, how often have you been bothered by any of the following problems? *[Matrix scale]*

Item	Not at all	Several days	More than half the days	Nearly every day
Little interest or pleasure in doing things	○	○	○	○
Feeling down, depressed, or hopeless	○	○	○	○

Item	Not at all	Several days	More than half the days	Nearly every day
Trouble falling asleep, staying asleep, or sleeping too much	○	○	○	○
This is an attention check item. Please select 'Nearly every day'	○	○	○	○
Feeling tired or having little energy	○	○	○	○
Poor appetite or overeating	○	○	○	○
Feeling bad about yourself — or that you are a failure or have let yourself or your family down	○	○	○	○
Trouble concentrating on things, such as reading the newspaper or watching television	○	○	○	○
Moving or speaking so slowly that other people could have noticed, or the opposite — being so fidgety or restless that you were moving around more than usual	○	○	○	○
Thoughts that you would be better off dead or of hurting yourself in some way	○	○	○	○

68. The following questions ask how you try to cope when facing difficulties in life. Please read each statement and select the option that best reflects how often you use each coping strategy.
[Matrix scale]

Statement	I have never done this	I do this occasionally	I do this about half the time	I always do this
I concentrate my efforts on doing	○	○	○	○

Statement	I have never done this	I do this occasionally	I do this about half the time	I always do this
something about the situation I am facing				
I get emotional support from others	○	○	○	○
I give up trying to deal with the situation	○	○	○	○
I take action to try to make the situation better	○	○	○	○
I get help and advice from other people	○	○	○	○
I try to think of strategies about what to do	○	○	○	○
I get comfort and understanding from someone	○	○	○	○
I give up trying to cope	○	○	○	○
I try to get advice or help from other people about what to do	○	○	○	○
I think hard about what steps to take next to deal with the situation	○	○	○	○

69. Based on the past month, please select the option that best describes you for each statement below. *[Matrix scale]*

Statement	Never	Rarely	Sometimes	Often	Always
I am able to adapt to change	○	○	○	○	○
I tend to recover quickly after hardship or illness	○	○	○	○	○

70. Over the past 2 weeks, how often have you been bothered by the following problems? *[Matrix scale]*

Item	Not at all	Several days	More than half the days	Nearly every day
Feeling nervous, anxious, or on edge	○	○	○	○
Not being able to stop or control worrying	○	○	○	○
Worrying too much about different things	○	○	○	○
This is an attention check item. Please select 'Nearly every day'	○	○	○	○
Trouble relaxing	○	○	○	○
Being so restless that it is hard to sit still	○	○	○	○
Becoming easily annoyed or irritable	○	○	○	○
Feeling afraid as if something awful might happen	○	○	○	○
