# Supplementary material for: Evaluation of a Digital Intervention for Monitoring and Improving Medication Adherence Among Real-World e-Consumers of HIV Preexposure Prophylaxis in China: Protocol for a Randomized Controlled Trial
Source: JMIR Res Protoc. 2026 Jun 29;15:e92750. doi: 10.2196/92750 (PMC13365895; doi:10.2196/92750)
Supplement: Multimedia Appendix 3 [file resprot_v15i1e92750_app3.pdf]

国家自然科学基金资助项目批准通知

（包干制项目）

罗思童　先生/女士：

根据《国家自然科学基金条例》、相关项目管理办法规定和专家评审意见，国家自然科学基金委员会（以下简称自然科学基金委）决定资助您申请的项目。项目批准号：82404326，项目名称：基于生态瞬时评估法的互联网购买PrEP人群服药依从性实时监测及精准干预研究，资助经费：30.00万元，项目起止年月：2025年01月至 2027年 12月，有关项目的评审意见及修改意见附后。

请您尽快登录科学基金网络信息系统（https://grants.nsfc.gov.cn），**认真阅读《国家自然科学基金资助项目计划书填报说明》并按要求填写《国家自然科学基金资助项目计划书》（以下简称计划书）**。对于有修改意见的项目，请您按修改意见及时调整计划书相关内容；如您对修改意见有异议，须在电子版计划书报送截止日期前向相关科学处提出。

请您将电子版计划书通过科学基金网络信息系统（https://grants.nsfc.gov.cn）提交，由依托单位审核后提交至自然科学基金委。自然科学基金委审核未通过者，将退回的电子版计划书修改后再行提交；审核通过者，打印纸质版计划书（一式两份，双面打印）并在项目负责人承诺栏签字，由依托单位在承诺栏加盖依托单位公章，且将申请书纸质签字盖章页订在其中一份计划书之后，一并报送至自然科学基金委项目材料接收工作组。纸质版计划书应当保证与审核通过的电子版计划书内容一致。**自然科学基金委将对申请书纸质签字盖章页进行审核，对存在问题的，允许依托单位进行一次修改或补齐。**

向自然科学基金委提交电子版计划书、报送纸质版计划书并补交申请书纸质签字盖章页截止时间节点如下：

1. **2024年9月9日16点：** 提交电子版计划书的截止时间；

2. **2024年9月16日16点：** 提交修改后电子版计划书的截止时间；

3. **2024年9月23日：** 报送纸质版计划书（一式两份，其中一份包含申请书纸质签字盖章页）的截止时间。

4. **2024年10月8日：** 报送修改后的申请书纸质签字盖章页的截止时间。

请按照以上规定及时提交电子版计划书，并报送纸质版计划书和申请书纸质签字盖章页，逾期不报计划书或申请书纸质签字盖章页且未说明理由的，视为自动放弃接受资助；未按要求修改或逾期提交申请书纸质签字盖章页者，将视情况给予暂缓拨付经费等处理。

附件：项目评审意见及修改意见表

国家自然科学基金委员会
2024年8月23日

附件：项目评审意见及修改意见表

项目批准号	82404326	项目负责人	罗思童	申请代码1	H3009
项目名称	基于生态瞬时评估法的互联网购买PrEP人群服药依从性实时监测及精准干预研究				
资助类别	青年科学基金项目		亚类说明		
附注说明					
依托单位	清华大学				
直接费用	30.00 万元		起止年月	2025年01月 至 2027年12月	

通讯评审意见:

<1>具体评价意见:

一、请评述该申请项目是否面向经济社会发展需要或国家需求背后的基础科学问题。请详细阐述判断理由。

该申请项目面向HIV感染暴露前预防服药依从性问题，对艾滋病防控十分重要，但是该项目是一项应用性研究，所涉及的方法--生态瞬时评估法是已有的，并且研究内容中并未对其有任何改进的相关内容，因此，项目基本未涉及基础科学问题。

二、请评述申请项目所提出的科学问题的创新性与预期成果的科学价值。

鉴于上述理由，本项目基本未涉及基础科学问题，基础科学创新性不足。预期成果可能有一定实用价值，但缺乏科学价值。

三、请评述申请人的创新潜力与研究方案可行性；如有可能，请对完善研究方案提出建议。

申请人接受过良好的科研训练，在传染病防控流行病学、管理、干预措施等领域取得了较多的成果，具备良好科研创新能力，但是研究基本未涉及基础科学前沿。按研究设计的研究内容，研究方案是节本可行的。建议申请自然科学基金时将研究聚焦在方法学创新等基础科学创新上。

四、其他建议

请注意核查作者署名的准确性。

<2>具体评价意见:

一、请评述该申请项目是否面向经济社会发展需要或国家需求背后的基础科学问题。请详细阐述判断理由。

该项目针对艾滋病高危人群PrEP服药依从性，以生态瞬时评估法（EMA）结合互联网，研发PrEP服药依从性实时监测和精准干预方法，为促进高危人群PrEP规范化使用、最大程度发挥PrEP预防HIV感染提供了新技术新手段。

二、请评述申请项目所提出的科学问题的创新性与预期成果的科学价值。

我国真实世界中PrEP用户服药依从性较差，尤其是其中按需服药用户，该项目以此为锲机，采用EMA，以数字健康技术为载体，开发服药依从性实时监测及精准干预方法和智能技术平台，在方法学上具有一定的新颖性。

三、请评述申请人的创新潜力与研究方案可行性；如有可能，请对完善研究方案提出建议。

申请人前期已开展并建立了互联网PrEP购药队列，获得了一些初步的实验结果，这为该项目的后续深入研究奠定了深厚的基础；同时申请人也在该项目相关领域取得了较好的研究成果，这也保障了该项目能够顺利达到预期目标。

四、其他建议

<3>具体评价意见:

一、请评述该申请项目是否面向经济社会发展需要或国家需求背后的基础科学问题。请详细阐

述判断理由。<br>该项目针对我国艾滋病重点人群进行PrEP研究，为实时检测准确干预PrEP服药依从性提供新方法，为我国重点人群艾滋病防控工作提供技术支撑，具有较好的科学性和公共安全防治价值。<br><br>二、请评述申请项目所提出的科学问题的创新性与预期成果的科学价值。<br>该项目研究并评价PrEP服药依从性实时监测及精准干预方法和智能平台，研究内容重点突出，方法具有较好的创新性。<br><br>三、请评述申请人的创新潜力与研究方案可行性；如有可能，请对完善研究方案提出建议。<br>该项目基于前期建立的PrEP网购者队列，有利于项目的顺利开展。针对该项目的科学假说，有着较好的前期研究支持。申请人科研背景较好。<br><br>四、其他建议
修改意见:<br><br><br><br>医学科学部<br><br>2024年8月23日
